# Laboratory cryo x-ray microscopy for 3D cell imaging

**DOI:** 10.1038/s41598-017-13538-2

**Published:** 2017-10-18

**Authors:** Emelie Fogelqvist, Mikael Kördel, Valentina Carannante, Björn Önfelt, Hans M. Hertz

**Affiliations:** 1 Department of Applied Physics, KTH Royal Institute of Technology/Albanova, 106 91 Stockholm, Sweden; 2 Department of Microbiology, Tumor and Cell Biology, Karolinska Institutet, 171 77 Solna, Sweden

## Abstract

Water-window x-ray microscopy allows two- and three-dimensional (2D and 3D) imaging of intact unstained cells in their cryofixed near-native state with unique contrast and high resolution. Present operational biological water-window microscopes are based at synchrotron facilities, which limits their accessibility and integration with complementary methods. Laboratory-source microscopes have had difficulty addressing relevant biological tasks with proper resolution and contrast due to long exposure times and limited up-time. Here we report on laboratory cryo x-ray microscopy with the exposure time, contrast, and reliability to allow for routine high-spatial resolution 3D imaging of intact cells and cell-cell interactions. Stabilization of the laser-plasma source combined with new optics and sample preparation provide high-resolution cell imaging, both in 2D with ten-second exposures and in 3D with twenty-minute tomography. Examples include monitoring of the distribution of carbon-dense vesicles in starving HEK293T cells and imaging the interaction between natural killer cells and target cells.

## Introduction

X-ray methods are emerging as a prime candidate for three-dimensional (3D) nano-imaging of intact cells in their native or near-native state. X-rays have the necessary absorption and scattering properties for such 3D imaging of thick objects while classical techniques have difficulty examining nanometer-scale detail in the unperturbed context of the full biological system: Electron microscopy and scanned-probe methods require thin samples or surfaces, respectively. Far-field optical microscopes^1^ lack the resolution while super-resolution optical microscopies^2^ (STED, PALM etc) have intrinsic difficulties providing 3D imaging with reasonable exposure times. However, present x-ray microscopes are typically based on synchrotron-radiation sources to provide sufficient flux for short exposure time, making them less accessible than the non-x-ray laboratory instruments discussed above. Here we report on laboratory cryo x-ray microscopy with the exposure time, contrast, and reliability to allow for routine high-spatial resolution 3D imaging of processes in intact cells and cell-cell interactions.

The two major methods for 3D x-ray imaging of intact cells are lens-based soft x-ray microscopy^3^ and lens-less hard x-ray methods based on coherent diffraction imaging (CDI)^4,5^. The CDI methods, which potentially have a dose advantage by avoiding lenses, presently typically claim 40–100 nm resolution with acceptable dose on cryofixed eukaryotic cells^6^ and algae^7^. Lens-based x-ray microscopes show better resolution on cryofixed hydrated cells and has also demonstrated many relevant biological results. Both methods presently rely on large-scale accelerator-based x-ray facilities, synchrotrons or free-electron lasers.

Lens-based soft x-ray microscopy in the water-window region (λ≈2.3–4.4 nm, E = 284–540 eV) allows high-resolution imaging of intact, thick hydrated samples with natural contrast. The basic idea is to use the large natural difference in absorption between proteins and lipids (i.e., carbon) and water (i.e., oxygen) in the water window for contrast while the short wavelength allows for far-field imaging with high resolution. Synchrotron-based microscopes were early to demonstrate that 3D water-window microscopy of cryofixed cells (x-ray cryo-tomography) allowed detailed visualization of subcellular organelles in relevant biological material^8–10^. In recent years several quantitative biological studies have delivered significant biological insight^11–14^. As in electron microscopy, cryofixation is essential for mitigating dose damage. Presently, a few synchrotron radiation facilities house soft x-ray cryo microscopes.

Here we demonstrate laboratory water-window x-ray microscopy with high resolution and high contrast on cryofixed cells with routine 10 s exposure time in 2D imaging and twenty-minute exposure time for 3D tomography. Such exposure times and reliability are prerequisites not only for enabling the tomographic 3D imaging but also to allow investigations on realistic biological samples, which typically are large and often heterogeneous, such as in the examples discussed below. The resolution is down to 50 nm half-period in the 2D and 100 nm half-period in the 3D. Previous laboratory x-ray microscopes provided imaging with similar resolution but with exposure times of a few hours^15^ for 3D and typically few minutes for 2D^16^, although noisy images could occasionally be recorded in 10 s^17^. The combination of long exposure times and low reliability essentially prohibited work on relevant biological samples. The improvement demonstrated in the present paper is due to improved source performance and higher-efficiency condenser optics. As for the sources, laboratory water-window microscopy has been performed with gas discharges and laser plasmas. The nitrogen pinch discharge typically operates at average line brightness of 4 × 10^9^ ph/(s × mm^2^ × mrad^2^ × line) at the λ = 2.88 nm line^18^, while the laser plasmas have demonstrated reliable operation at 4 × 10^10^ ph/(s × mm^2^ × mrad^2^ × line) at the λ = 2.48 nm using liquid-nitrogen-jet target^19^. Martz *et al.* ^17^ demonstrated that this source could produce > 1.5 × 10^12^ ph/(s × mm^2^ × mrad^2^ × line)  and we have recently stabilized the liquid jet to allow for long-term operation at these brightness levels^20^. The fabrication of normal-incidence condenser optics has reached a new level of perfection the last few years, typically increasing the average reflectivity from 0.6% to over 4.5%.

The improved laboratory cryo x-ray microscope provides a new laboratory window for high-resolution and high-contrast observations of cell-biological processes in 2D and 3D. We demonstrate the improved system performance on two biological topics, monitoring the development and distribution of carbon-dense vesicles in starving cells and imaging the interaction between natural killer cells and target cells.

Nutrient starvation causes cellular stress that may induce autophagy^21,22^. This and other important cellular degradation pathways involve the formation and transformation of several types of µm and sub-µm vesicles in the cytoplasm. Determining the distribution and development of such vesicles by imaging the unstained cell is intrinsically difficult with classical methods^23,24^ but has been observed with synchrotron-based soft x-ray microscopy^25^. Here we show that the improved laboratory x-ray microscope can image these often carbon-dense vesicles in unstained starving HEK293T cells in both 2D and 3D with significant contrast, allowing for quantitative analysis of the vesicle development.

Natural killer (NK) cells are part of the innate immune system and kill target cells in a carefully regulated process^26–28^. The detailed molecular steps of the process, the formation of the NK-cell-target-cell immune synapse^27^, have been investigated with confocal microscopy as the primary imaging tool, recently complemented by super-resolution imaging^29^ while the immune synapse between T-cells and dendritic cells has been imaged with synchrotron-based soft x-ray microscopy^14^. Here we show that the improved laboratory x-ray microscope allows 2D and 3D imaging of the interaction between NK cells and HEK293T target cells.

## Results

### Short-exposure laboratory water-window cryo microscopy

**Figure 1:**
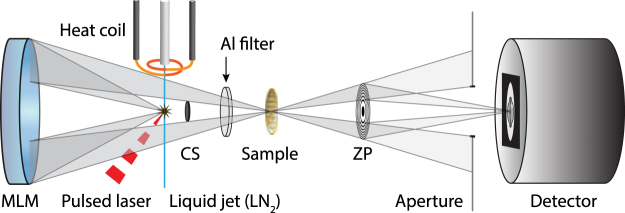
Short-exposure laboratory water-window cryo microscopy. The Stockholm laboratory x-ray microscope is based on a liquid-nitrogen-jet laser-plasma source producing 2.48 nm line emission. A normal-incidence multilayer condenser mirror (MLM) focuses the 500 eV x-rays onto the sample, which is imaged by the zone plate (ZP) onto the CCD detector. A central stop (CS) creates a hollow-cone illumination and a 200 nm aluminum filter absorbs scattered visible light while transmitting the soft x-rays. The heating coil around the nozzle tip is a recently added feature to improve the liquid nitrogen jet stability.

Figure 1 shows the laboratory cryo x-ray microscope. In its basic arrangement it consists of a liquid-nitrogen-jet laser-plasma source, a normal-incidence multilayer mirror as condenser, a cryo sample stage, a Ni zone plate, and a backillumated CCD detector, all in vacuum^15^. In the present experiments the liquid-jet source is powered by a 100 W 2 kHz 600 ps diode-pumped Nd:YAG slab laser to produce narrow-bandwidth λ = 2.48 nm line emission and a 30 nm outer zone width 200 μm diameter Ni zone plate was used for the imaging. The longer focal length (2.42 mm at λ = 2.48 nm) provides enough space between sample holder and zone plate not to limit the tilt range for the tomographic recordings. The magnification M is 770 × resulting in 17.5 nm pixels in the object plane from 13.5 µm detector pixels. More details are given in Methods.

The key hurdle for reaching the microscope performance demonstrated here has been the source. Jet instabilities have lowered reliability, uptime and average brightness. Here we have integrated the radiation heating stabilization of ref.^20^ in the microscope as well as a flash-imaging system allowing the continuous monitoring of the jet with ~5 µm spatial resolution and 4 ns temporal resolution. With these means the laser-plasma can be moved further away from the nozzle orifice and thereby create less disturbance to the jet and a more stable source emission. Despite that we typically operate the laser at lower power than in ref.^17^, the average λ = 2.48 nm brightness is higher or similar and, more importantly, stable over hours. This improvement is combined with recent advances in normal-incidence multilayer mirrors. The new Cr/V condenser mirror has an average reflectivity of 4.66%, as compared with our previous Cr/V mirror of 0.6%. In total, this results in an improvement of approximately 8 times in the flux at the sample compared our previous arrangements, reducing the normal-incidence 2D exposure time to the 10-s range. In addition, the improved reliability of the stabilized source is essential for working with relevant cell-biological samples and for the multiple exposures necessary for tomography. As for the spatial resolution, Fourier Ring Correlation analysis (cf. Methods) of all 2D images presented below result in 50–100 nm half-period resolution. A more detailed analysis of the spatial resolution of the imaging system is found in the Discussion.

### 2D x-ray microscopy of starving HEK293T cells

**Figure 2:**
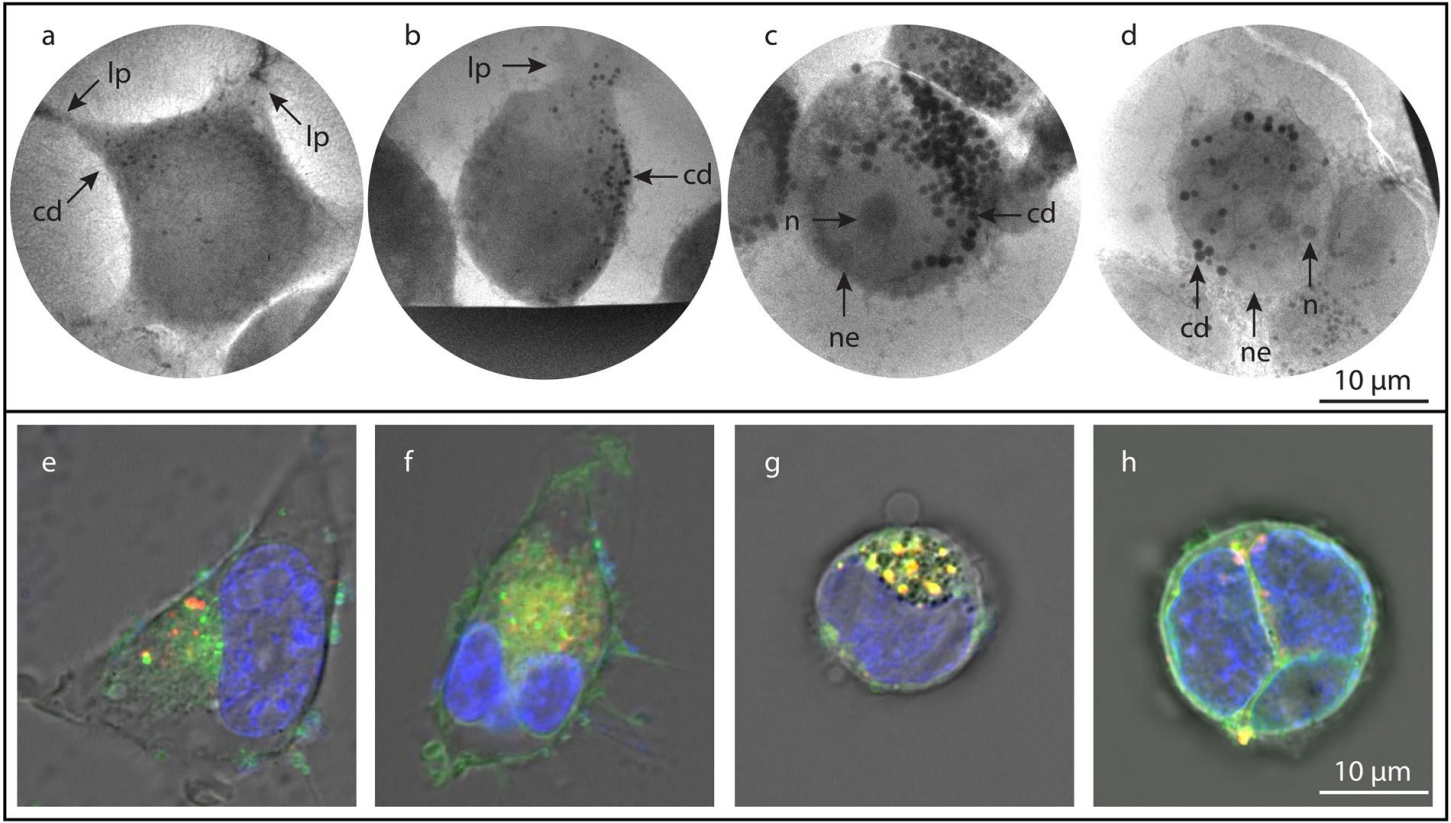
HEK293T cells in different stages of starvation. **(a–d)** Laboratory x-ray absorption microscopy of HEK cells with increasing starvation. **(a)** Slightly starved cell with lamellipodia (lp) spread across the carbon grid and few and small (0.37 µm diam) carbon-dense vesicles (cd). **(b)** Early-stage starvation results in rounding of cell shape, decreasing adhesion and lamellipodia, and growth in number of carbon-dense vesicles. **(c)** Increasing starvation results in strong increase in number as well as diameter (0.88 µm diam) of carbon-dense vesicles. **(d)** A highly starved HEK 293 T cell showing a few nuclear envelopes (ne), nucleoli (n), and large carbon-dense vesicles. **(e–h)** Confocal imaging of HEK293T cells at approximately same stages of starvation, for comparison. Here the cells have been stained for nucleus (blue), membranes (green) and acidic organelles (red).The fluorescent images are overlaid on the grey DIC image. Scale bars 10 µm.

Figure 2a–d show 10-s exposure cryo x-ray microscopy of human embryonic kidney cells (HEK 293 T) in increasing stages of starvation. In Fig. 2a a slightly starved cell (less than 1 day of starvation) still appears healthy with stretched-out lamellipodia (marked lp) adhering to the carbon film. The cell shows 25–30 small (diameter 0.37 ± 0.06 µm) carbon-dense vesicles (cd), sparsely distributed in the cytoplasm. As starvation increases (Fig. 2b, 1–2 days of starvation) adhesion and lamellipodia decrease, cell shape changes, and the number and size of the carbon-dense vesicles increase (50–55 and 0.44 ± 0.07 µm, respectively). In Fig. 2c the process has gone further (approx. 2 days of starvation) and we count 80–90 large (0.88 ± 0.15 µm) carbon-dense vesicles. Figure 2d, finally, shows a cell starved approx. 3 days to the point of what appears to be close to cell death. Here the cell has changed its shape and it contains multiple large carbon-dense vesicles(up to 1.3 μm). We also note clearly observable nuclear envelopes (ne) surrounding what appear to be multiple nuclei. Several nucleoli (n) are also clearly visible in the nuclei. The nucleoli are clearly distinguishable from the carbon-dense vesicles by changing the focus, which is done for every image to obtain best focus.

In Fig. 2e–h we depict fluorescence confocal microscopy of the same HEK293T cell-line at similar stages of starvation, for comparison with the x-ray microscopy. Here nuclei are stained Hoechst NucBlue (blue), membranes with DiO (green), and acidic vesicles with LysoTracker (red). The laboratory x-ray microscopy images agree well with the confocal microscopy. As the starvation progresses, a similar change in cell shape, adhesion, and lamellipodia protrusion is observed in the confocal images as is the general trend that the membrane-bound and/or acidic vesicles grow larger and more numerous. Such vesicles are an important part of the autophagic process^21^. It is interesting to note that the x-ray microscopy images provide more details as regards, e.g., number and size of the vesicles despite that no staining is used. This quantitative information was not possible to retrieve from our confocal images.

### 3D x-ray tomography of a starving HEK293T cells

**Figure 3:**
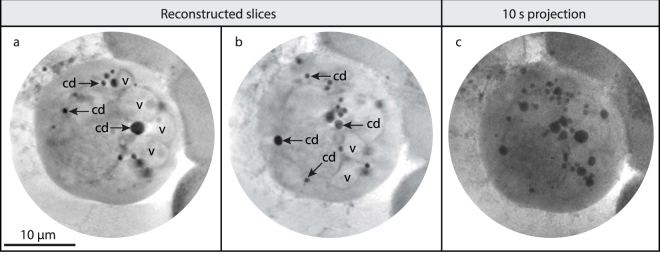
3D tomographic reconstruction of a starving HEK 293T cell. (**a,b)** Two slices through the 3D reconstructed cell volume. Vacuoles (v) and carbon-dense vesicles (cd) are marked in the image. The full volume and a segmentation are given in Supplementary Material. **(c)** One of the 2D 10-second projections used for the tomography, for comparison. Scale bar 10 µm.

The 3D volume image of a highly starved HEK293T cell was reconstructed from a tomographic dataset with 69 projections over a tilt range of 102° with 1.5° angular increments (cf. Methods). Figure 3a and b show two slices separated by 2 µm through the reconstructed cell volume. In both slices carbon-dense vesicles (cd) and what appears as several vacuoles (v) are clearly visible. The vacuole membranes are clearly observed in the slices, although they are too thin to be fully resolved. Figure 3c shows one of the 2D projections, for comparison. It is clear that the tomography provides better contrast in addition to the obvious benefit of full 3D localization information. The full 3D data stack and an example of segmentation are shown in Supplementary Material.

### 2D x-ray microscopy of NK-cell target-cell interaction

**Figure 4:**
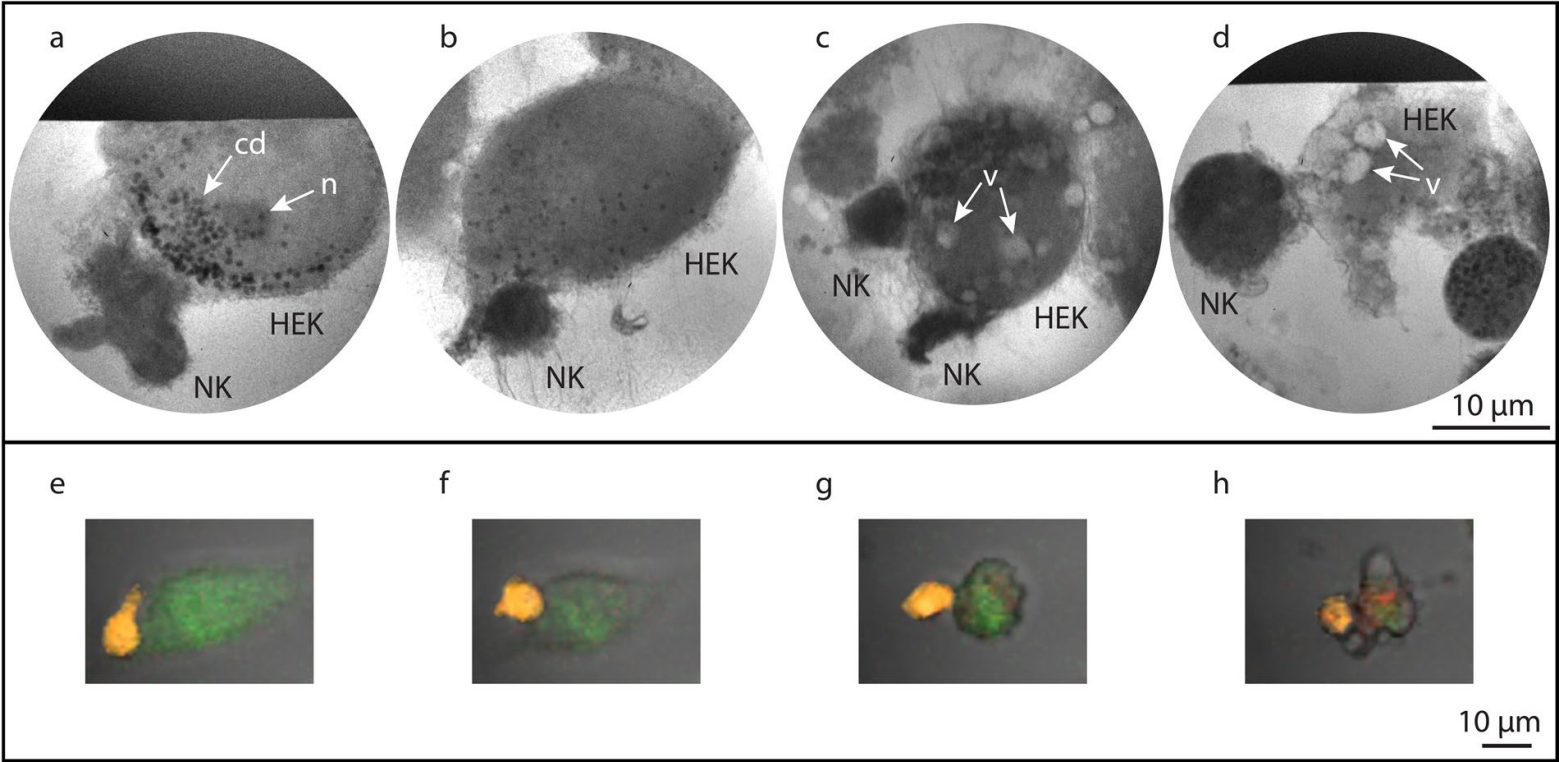
2D x-ray microscopy of the interaction of an NK cell with a HEK target cell. (**a–d)** X-ray microscopy images obtained with 10 s exposure time showing different stages of NK-target cell interaction, from **(a)** initial adhesion, **(b)** over firm adhesion, **(c)** starting apoptosis and **(d)** target cell death. **(e–h)** Snap-shots from a time-lapse sequence by confocal microscopy of an NK cell forming an immune synapse and killing a HEK target cell. The NK cell is stained orange and the HEK target cell is stained green and red. Transition from green to red indicates cell death. Scale bars 10 µm.

Figure 4a–d show 10-s exposure cryo x-ray microscopy of NK-cell interaction with HEK293T cells at four different stages of the killing process. Figure 4a shows an NK cell loosely adhered to the target cell while Fig. 4b and c depict NK cells firmly adhered to the target cells. The target cell in Fig. 4c has rounded-up, and appears to have a diffuse cell border as well as several vacuoles (v), possibly induced by an NK-mediated cytolytic attack^27,30^. Figure 4d shows an NK cell in loose contact with a dead target cell, possibly captured during the detachment phase^27^ after NK-mediated killing of the target. Also here we note the formation of vacuoles (v). In all four images a pattern of a partially unresolved filament structure at the NK-target cell interface is observed.

For comparison, Fig. 4e–h show confocal scanning microscopy of the corresponding stages, from the formation of the immune synapse to the target cell death. Here the NK cell is marked with Calcein Red-Orange (orange) and the target cell is marked with Calcein Green (green) and DDAO-SE Far-Red (red).

### 3D x-ray tomography of NK-cell target-cell interaction

**Figure 5:**
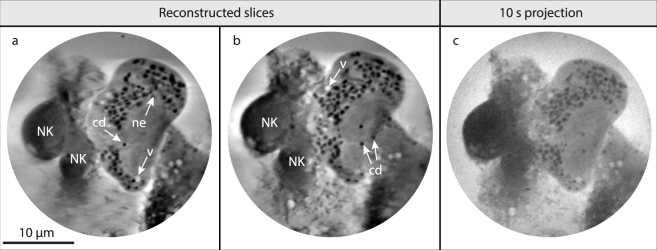
3D tomographic reconstruction of NK cell interaction with starving HEK293T cell. (**a,b)** Two slices from the reconstructed 3D volume of two NK cells interacting with a starved HEK293T cell. Vacuoles (v), nuclear envelope (ne), and carbon-dense vesicles (cd) are marked. The full volume and a segmentation are provided in Supplementary Material. **(c)** One of the 2D 10-second projections used for the tomography, for comparison. Scale bar 10 µm.

The 3D volume of two NK cells interacting with a starved HEK293T cell was reconstructed from a tomographic dataset with 75 projections over a tilt range of 111° with 1.5° angular increments (cf. Methods). Figure 5a and b show two slices located 0.7 μm apart in the 3D volume with vacuoles (v), nuclear envelope (ne), and carbon-dense vesicles (cd) clearly distinguishable. From browsing through the volume (full stack, see Supplementary Material), it is clear that all carbon-dense vesicles are located outside the nucleus of the HEK 293T cell. Figure 5c shows a 10 s exposure time projection image, for comparison. In Supplementary Material another reconstruction of NK-HEK interaction is depicted, probably at a somewhat later stage in the killing process given the rounded target cell with a vacuolated appearance. An example of segmentation is also presented in Supplementary Material.

## Discussion

We have demonstrated that laboratory cryo x-ray microscopy can be routinely employed for 2D and 3D high-resolution high-contrast imaging of intact biological structures in unstained cellular samples in their near-native (cryofixed) environment with short exposure times and high reliability. The key advances to the microscope enabling this capacity include improvements in source stability, source reliability, condenser reflectivity in combination with sample preparation. This allows laboratory x-ray microscopy to investigate realistic biological samples and the system is demonstrated by monitoring the development and distribution of carbon-dense vesicles and morphological changes in starving cells and imaging the interaction between natural killer cells and target cells.

In the starving HEK cells of Figs 2 and 3 we show that the unstained x-ray imaging of the cell development is consistent with the parallel confocal imaging as regards all the major structural factors such as cell shape, withdrawal of lamellipodia, and formation of vacuoles and vesicles. Of particular interest is the demonstrated possibility to quantitatively monitor the growth in number as well as in size of the carbon-dense vesicles. Although our present arrangement does not have the molecular sensitivity to designate these carbon-dense vesicles to be part of an autophagic process, the results suggest that the laboratory x-ray microscope should be applicable for quantitative autophagy studies, albeit after being combined with fluorescence microscopy for direct correlation of molecular imaging with the x-ray imaging of the same samples^13^.

Also for the NK-cell target-cell imaging, the unstained x-ray imaging of the killing process compares well with the confocal imaging, as regards cell shape, membrane attachment, and final disintegration of the target cell. Here the possibility to image the immune synapse in unstained NK-target-cell complexes in 3D may become a powerful tool. Conventional techniques for optical microscopy, e.g., wide-field and confocal microscopy, suffer from lower spatial resolution and electron microscopy requires tedious sample preparation. A better understanding of the observed but unresolved filament structure in the interface as well as the observed vacuolization process would be of large importance. Such studies would, however, require further improvements of the x-ray microscope performance, especially as regards resolution and contrast.

The spatial resolution as assessed by FRC was 50–100 nm half-period in all 2D x-ray images presented here. Better matching of zone plate parameters with source bandwidth (cf. Methods) and a reduction of minor residual vibrations could improve this. In the 3D images the reconstructed tomographic slices presented here show features down to the 100 nm-range in the central parts of the sample, while being more blurred towards the outer parts. The main reasons are the limited depth of focus (DOF) of the Fresnel zone plates and the missing wedge in the tomographic data set. These problems are inherent for all x-ray microscopes when using a TEM-type cryo sample stage, which is the standard arrangement for studying adherent cells or cell interactions. Naturally, smaller cells like algae and/or cell types that accept capillary sample stages^8^ avoid this problem. As for the DOF problem Selin^31^ has proposed a method, focus-stack back-projection, which would be helpful. The missing wedge is in practice determined by the shadowing of highly absorbing features, neighboring cells, and the ice layer thickness. A specialized sample holder with lower bars and smaller diameter should help. Also the recently invented dual-axis tomography at the Alba microscope reduces this problem^32^. Contrast may be improved by increasing the partial coherence^9^. Increasing coherence has previously not been possible at laboratory x-ray microscopes since this intrinsically decreases the photon flux, making exposure times prohibitively long. With the improved source and condenser arrangement, this may be an alternative.

## Methods

### Laboratory water-window cryo x-ray microscopy

Figure 1 shows the experimental arrangement, which builds on previous versions of the Stockholm laboratory x-ray microscope^15^. The water-window λ = 2.48 nm (E = 500 eV) source is generated by focusing the λ = 1064 nm beam of a 2 kHz, 600 ps diode-pumped Nd:YAG slab laser (Inst. f. Lasertechnik, FHG Aachen) typically operated at 80–100 W onto a 30 µm diameter 40–60 m/s liquid nitrogen jet^17,19^. The stability of the jet is recently improved by a radiative heating arrangement^20^. In parallel, the jet position is continuously camera-monitored with an integrated flash-illumination system consisting of a fluorescent dye excited by a 4 ns, λ = 532 nm, 4 W, 20 Hz Nd:YAG laser providing ~5 µm spatial resolution. These two measures allow the operation at a larger distance (typically 3.5 mm) from the nozzle orifice providing the necessary long-term reliability and stability for microscopy in both 2D and 3D of relevant biological samples.

The typically 30 µm diameter plasma source is imaged with 1.6 × magnification onto the sample by a 58 mm diameter Cr/V multilayer condenser mirror (radius of curvature 350 mm) (Optix fab, Jena). This 500 double-layer, λ/Δλ = 450 bandwidth, and R = 4.66% average reflectivity mirror selects the λ = 2.48 nm hydrogen-like (NVII) line emission for monochromatic illumination of the sample. A 200 nm Al filter blocks scattered light and a central stop produces the hollow-cone illumination. The sample stage used in the microscope is a modified TEM goniometer stage (FEI) that allows movement in all spatial dimensions, including tilt along one axis. The samples are prepared on commercial holey carbon Au TEM grids, mounted on a temperature regulated custom-made cryo sample holder (Gatan) and inserted into the sample stage.

A 30 nm outer zone width, 200 µm diameter Ni zone plate images the samples onto the CCD. The choice of zone plate parameters reflects a balance between the need for large working distance for the tomography while still avoiding the influence of chromatic aberrations on the resolution. This long-working-distance objective (*f* = 2.42 mm at λ = 2.48 nm) allows ±90° tilt range of the custom cryo sample holder. However, the intrinsic large number of zones (N = 1667) may start introducing chromatic aberrations given the estimated source band width of λ/Δλ ≈ 700–1000^33^.

The images are recorded by a cooled back-illuminated CCD (iKon-L, Andor) with 2048 × 2048 13.5 µm pixels. All experiments were performed at a magnification M = 770, resulting in a pixel size of 17.5 nm in the sample plane and a field of view (FOV) of 35.9 µm, large enough to fit several cells in one image and to search for interesting specimen over large sample areas.

### Sample preparation for x-ray microscopy

The HEK cells (cf. below) were seeded (~10^5^ cells/well) and cultivated on carbon Au TEM grids in 4-well plates under physiological conditions (37 °C and 5% CO_2_). For the carbon-dense vesicle visualization, the cells were imaged after one to three days of starvation, i.e., no nutrients were added. We note that the number of starved days is a very approximate indication of the cell’s stage of starvation since it also depends on several other parameters, such as number of cells, volume of medium etc. For the NK-target cell experiments, ~5 × 10^5^ primary IL-2 activated NK cells (cf. below) were added to each well after 24 h incubation. The abundance of NK cells was to guarantee multiple interactions on the grid. Most of the non-interacting NK cells were removed by blotting.

The grids were blotted to remove excess liquid and plunge-frozen in liquid ethane. To ensure a thin amorphous ice layer, we monitored that the liquid surface was concave, indicating a thickness smaller than the height of the gold bars (10 µm). The grids were then transferred to the microscope and kept at −170 °C throughout the experiments on the custom cryo sample holder.

### Data acquisition

The grid was first searched for an interesting specimen with 1 s exposures and then the 2D imaging was done with 10 s exposures. The photon flux is approximately 160 ph/pixel (per 10 s) with no object (i.e., flat-field recordings) and 60 ph/pixel with a typical sample amorphous ice layer (corresponding to 8 μm ice). The dose for a 10 s exposure is estimated to 10^5^ Gy.

For the tomography we record approximately 70 projections, typically in a range of angles up to ±57°, where the upper limit is determined by shadowing of the sample by the grid bars or neighboring cells as well as reduced x-ray transmission through the slightly concave ice layer. To compensate for the latter, 10 s exposures were used for angles < ±30° and 20–30 s exposures for higher angles. Although the total exposure time for a tomography series is in the 20 min range, the total data acquisition time is in the 2-hour range due the still manual sample repositioning between exposures.

### Data processing and reconstruction

All 2D images are baseline corrected and normalized for the source movement and thickness variations in the ice-layer by dividing each image with a heavily blurred version of itself. The images were then smoothed by applying a Gaussian filter $(\frac{{r}^{2}}{2{\sigma }^{2}})$ with σ = 1.5, where r is in pixels.

The tomographic reconstruction was performed with the simultaneous iterative reconstruction algorithm (SIRT) in TomoJ^34,35^ with 50 iterations and relaxation coefficient 0.5. Projections were treated as the 2D images but with a σ = 2 Gaussian filter, reflecting the lower detail obtainable given the depth of field and limited tilt-range. Alignment of the projections were performed by manual correlation correction in TomoJ and refined using the automated landmark alignment. Images were binned 2 × 2 for faster reconstruction.

### Image analysis

The quantitative analysis (of, e.g., the carbon-dense vesicles) was done manually by two independent observers, whose results typically differed less than ± 5%, both in number and diameters. Given the low difference and high contrast, the microscope method demonstrated here should allow for automatic quantification in both 2D and 3D.

The resolution in all 2D x-ray images was assessed using Fourier ring correlation (FRC) with the standard ½-bit resolution threshold^7,36^. This threshold was also found to produce conservative values of the highest resolvable spatial frequencies when applied to images of a Siemens star.

### Human embryonic kidney (HEK) cell culture

The human embryonic kidney cells 293T (HEK293T) were cultured in RPMI-1640 (SH30027 Thermo Scientific, MA, USA) supplemented with 10% fetal bovine serum (SV30160; Thermo Scientific, MA, USA), 100 U ml^−1^ penicillin–100 mg ml^−1^ streptomycin, 1 × MEM Non-Essential Amino Acids Solution (Thermo Fisher Scientific, MA, USA), 1 mM sodium pyruvate, 2 mM l-glutamine (Sigma Aldrich, MO, USA) and maintained at 37 °C, 5% CO_2_. For confocal microscopy, HEK293T cells were cultured in 35 mm glass-bottom petri dishes (MatTek Corporation, MA, USA) for two days before the experiment.

### Natural killer (NK) cell isolation and culture

Peripheral blood mononuclear cells (PBMCs) were obtained from healthy donor buffycoats by Ficoll–Hypaque gradient separation (GE-Healthcare). NK isolation from PBMCs was performed by negative selection using the NK cell Isolation Kit (Miltenyi Biotec GmbH, GE) according to manufacturer’s instructions. Purity of enriched polyclonal NK cells was measured as percentage of CD56^+^ CD3^−^ population by flow cytometry and confirmed to be >95%. Purified polyclonal NK cells were maintained in RPMI 1640 supplemented with 10% FBS and 100 U/ml of r-IL-2 (R&D System Inc., MN, USA).

### Live cell confocal microscopy

Images were acquired using inverted laser scanning confocal microscopy (LSM 880 or LSM510 META, Zeiss, Germany) equipped with an environmental chamber maintaining the cells under physiological condition, using the laser lines 405 nm, 488 nm, 561 nm and 633 nm combined with tunable emission filters set to match the emission spectra of the used probes. For fluorescent staining, cells were washed, incubated with the appropriate dye according to the manufacturer’s instructions,washed, and used for experiments. Except for the staining, similar protocols were used for x-ray and confocal microscopy.

Figure 2 was acquired in single optical sections or Z-stacks of several optical sections (≈0.5 μm apart) with a 63×/1.4 oil immersion objective. The cells were stained with Hoechst 33342 NucBlue® Live ReadyProbes® Reagent (Thermo Fisher Scientific, MA, USA), DiO Cell-labeling solution (Thermo Fisher Scientific, MA, USA), and LysoTracker® Deep Red (Thermo Fisher Scientific, MA, USA) for nuclei, membranes and acidic organelles, respectively. Membrane-bound and acidic organelles are involved in the autophagic process^21,22^. The time-lapse sequence of NK-HEK interactions (Fig. 4) were performed with a 20×/0.3 objective acquiring one image every 2 min for 12 hours^37^. The NK cell was stained with CellTrace™ Calcein Red-Orange, AM (Thermo Fisher Scientific MA, USA) and the HEK cell with CellTrace™ Calcein Green, AM (Thermo Fisher Scientific MA, USA), and DDAO-SE Far-Red (Thermo Fisher Scientific MA, USA).

## Electronic supplementary material

Supplementary material

Video S3

Video S4

Video S5
