# Supplementary material for: Laboratory cryo x-ray microscopy for 3D cell imaging
Source: Sci Rep. 2017 Oct 18;7:13433. doi: 10.1038/s41598-017-13538-2 (PMC5647409; doi:10.1038/s41598-017-13538-2)
Supplement: Supplementary file 1 — Supplementary material [file 41598_2017_13538_MOESM1_ESM.pdf]

Supplementary Material

Laboratory x-ray cryo microscopy for 3D cell imaging.

Emelie Fogelqvist[1], Mikael Kördel[1], Valentina Carannante[2], Björn Önfelt[1,2], and Hans M. Hertz[1]*

[1] Department of Applied Physics, KTH Royal Institute of Technology/Albanova, 106 91 Stockholm, Sweden

[2] Department of Microbiology, Tumor and Cell Biology, Karolinska Institutet, 171 77 Solna, Sweden

*Correspondence: hertz@biox.kth.se

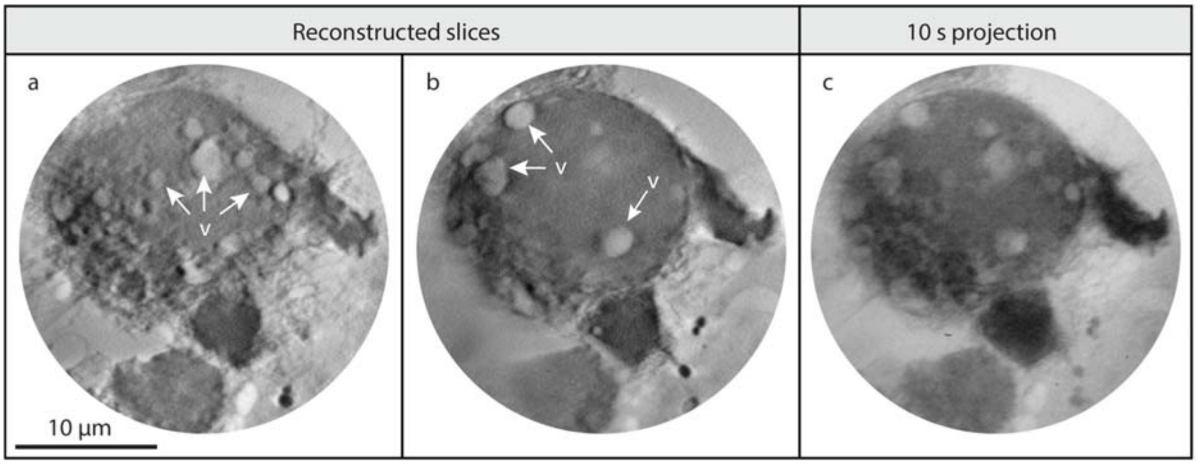

Figure S1. 3D tomographic reconstruction of NK cell interaction with HEK293T cell. This figure shows a similar interaction as in Fig. 5 but later in the killing process with vacuole formation. (**a-b)** Two slices from the reconstructed 3D volume of two NK cells interacting with a starved HEK293T cell. Vacuoles (v) are marked in the images. The full volume stack is provided in Supplementary material. **(c)** One of the 2D 10-second projections used for the tomography, for comparison. Scale bar 10 µm.

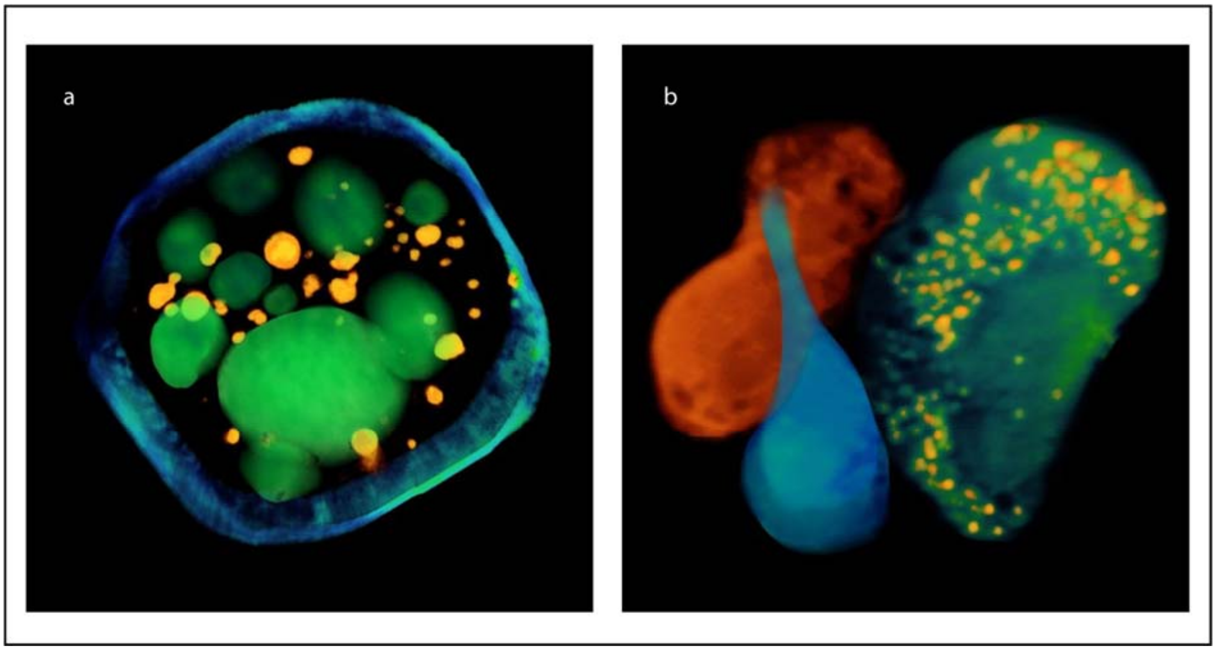

Figure S2. Volume segmentations. (a-b) The figure shows preliminary segmentations of the reconstructed volumes presented in Fig. 3 (Video S3) and Fig. 5 (Video S4). **(a)** depicts a segmentation of a starving HEK 293T cell showing carbon-dense vesicles (orange) vacuoles (green) and cell membrane (blue). **(b)** shows two NK cells (blue, orange) that are in close contact with a starved HEK293T cell containing carbon-dense vesicles (orange).

Video S3. Full 3D x-ray volume stack of Fig. 3, a starving HEK 293T cell. Each voxel corresponds to 35 nm cubed in the sample plane.

Video S4. Full 3D x-ray volume stack of Fig. 5, NK cell interaction with starving HEK 293T cell. Each voxel corresponds to 35 nm cubed in the sample plane.

Video S5. Full 3D x-ray volume stack of Fig. S1 NK cell interaction with HEK293T cell. Each voxel corresponds to 35 nm cubed in the sample plane.
